# Supplementary material for: ZAF, the first open source fully automated feeder for aquatic facilities
Source: eLife. 2021 Dec 9;10:e74234. doi: 10.7554/eLife.74234 (PMC8776251; doi:10.7554/eLife.74234)
Supplement: Supplementary file 2. — The table lists the necessary parts to build ZAF. Most of the parts are generic and can be replaced by components with similar specifications. [file elife-74234-supp2.docx]

| Components | Parts name | Supplier | Article Number | Number | Unite Price (USD) | Total Price  (USD) |
| --- | --- | --- | --- | --- | --- | --- |
| Frame  Servo & Food container  Pumps & Valve  Food Mixing  Safety  Tubing  Electronics | Makerbeam Starter Kit  Eheim Automatic feeding unit  Digital Servo  Magnets  Pumps  Valve DC 12V ¼”  Cable ties  Tube Holder  Funnel  Water sensor  Minipump  Type 1 tubing 3/16”  Type 2 tubing 4mmID, 6mm OD  Polycarbonate plastic sheet  40 aquarium connectors  Velcro strips  Wire connectors T-type  Wire connectors I-Type  Motor Drivers  Canakit Raspberry Pi 3B+  Servo Hat 16 Channel  LCD Touch Screen 1024X600 | Makerbeam  Eheim  N/A  Dymag  Trossen Robotics  Digiten  McMaster-Carr  Asayu  Karzone  DAOKI  Walfront  CNZ  NACX  Robosource  Asayu  Velcro  Biantie La  Biantie La  Qunqi  Canakit  Adafruit  Longruner | 103318  NA  DS318  3MM-mix  TI-TG-02B-DC12B  DC 12V ¼”  80005k2  N/A  N/A  TS-VS-292-CA  12V DC 6W  N/A  N/A  N/A  N/A  S-5751  N/A  N/A  L298N  N/A  2327  LSC7B | 1  1  1  1  4  1  1  1  1  1  1  1  2  1  1  1  2  2  2  1  1  1 | 112.25  23.47  16.66  14.99  24.95  7.49  25.05  5.99  6.99  20  15.49  5.99  22.88  24.99  11.99  37  9.98  9.99  8.69  69.99  17.5  56.99 | 112.25  23.47  16.66  14.99  99.8  7.49  25.05  5.99  6.99  20  15.49  5.99  45.76  24.99  11.99  37  19.76  19.98  17.38  69.99  17.5  56.99 |

**Total Price 675.10$**
