# Supplementary material for: ZAF, the first open source fully automated feeder for aquatic facilities
Source: eLife. 2021 Dec 9;10:e74234. doi: 10.7554/eLife.74234 (PMC8776251; doi:10.7554/eLife.74234)
Supplement: Supplementary file 3. — The table lists the necessary parts to build ZAF+. Most of the parts are generic and can be replaced by components with similar specifications. [file elife-74234-supp3.docx]

| Components | Parts name | Supplier | Article Number | Number | Unite Price (USD) | Total Price  (USD) |
| --- | --- | --- | --- | --- | --- | --- |
| Frame  Servo & Food container  Pumps & Valve  Food Mixing  Safety  Tubing  Electronics | Makerbeam Starter Kit  200mm black anodised Makerbeam  300mm black anodised Makerbeam  Makerbeam corner cube  Eheim Automatic feeding unit  Digital Servo  Magnets  12V DC Pumps  Solenoid valve quick connect ¼”  Check valve ¼”  Tube Holder  Funnel  Water sensor  Minipump  Soft Plastic tubing ⅜” 50ft  Soft Plastic tubing ¼” 100ft  Push to connect reducer  manifolds  T shape connectors  T shape connectors  Waterproof box  Screw terminal X8  Motor Drivers  Canakit Raspberry Pi 3B+  Solder Breadboard  LCD Touch Screen 1024X600  Arduino Mega 2560R3  16X module relay | Makerbeam  Makerbeam  Makerbeam  Makerbeam  Eheim  N/A  Dymag  Bayite  Digiten  Blulu  Asayu  Karzone  DAOKI  Walfront  McMaster  McMaster  McMaster  McMaster  McMaster  McMaster  Ogrmar  Milapeak  Qunqi  Canakit  Sparkfun  Longruner  Sparkfun  Sainsmart | 103318  10090  100102  100988  NA  DS318  3MM-mix  XX328  N/A  40141600  N/A  N/A  TS-VS-292-CA  12V DC 6W  5233k65  5233k52  5779k355  52045k216  51055k27  51055k195  N/A  N/A  L298N  N/A  PRT-12699  LSC7B  Dev-11061  170C20 | 1  2  2  1  4  1  1  2  30  1  1  1  1  1  1  3  1  3  2  1  1  1  4  1  1  1  2  2 | 112.25  12.5  9.75  16.95  23.47  16.66  14.99  29.99  7.49  7.99  5.99  6.99  20  15.49  49  18  7.30  26.64  4.41  4.41  34.99  12.49  8.69  69.99  12.49  56.99  39.65  20.99 | 112.25  25  19.5  16.95  93.88  16.66  14.99  59.98  224.7  7.99  5.99  6.99  20  15.49  49  54  7.30  79.92  8.82  4.41  34.99  12.49  34.76  69.99  12.49  59.99  79.3  41.98 |

**Total Price 1189.81$**
