# Supplementary material for: ZAF, the first open source fully automated feeder for aquatic facilities
Source: eLife. 2021 Dec 9;10:e74234. doi: 10.7554/eLife.74234 (PMC8776251; doi:10.7554/eLife.74234)
Supplement: Supplementary file 4. — This table provides solutions to common minor issues encountered during ZAFs construction and operations. [file elife-74234-supp4.docx]

| **Problem** | **Cause** | **Solution** |
| --- | --- | --- |
| - Burning of electronic components  (ZAF specific).  - Food distribution is non homogeneous  (ZAF specific).  - All the pumps connected to a motor controller (LN298) are not working.  - A lot of food remains in the food preparation tank.  - No food going inside the food preparation tank.  - Water leak at the pump output.  - Dirty fish water feeding .  - Tubes are getting dirty quickly.  - The mixed water/food is not going out of the tube  - Raspberry Pi is not responsive | - Voltage ripples due.  - tubing issues.  - Wiring problem.  - bad pumping.  - Food delivery is clogged.  - Tubing connections with the pump.  - Overfeeding.  - Cleaning is not well done.  - The pump is not running correctly and our tubes are too long. | - Add 1000uF capacitors in parallel to the motor terminals  - All the tubes should be same length  - One tube might be pinched or bent.  - Check the T splice connector wiring connections  - Check the tube position inside the food preparation container, it must be at the bottom.  - Possible pump malfunction, change the pump.  - Clean the funnel and the food dispenser output.  - Change the zip tie and eventually cut the tip of the damaged tube.  - Reduce the food quantity delivered by i. Adjusting the software, ii. Adjust the food dispenser closure.  - Add more cleaning programs.  - Possible pump malfunction, change the pump.  - Increase the pump running period in the code source. |
